# Inflammation in Cerebral Venous Thrombosis

**DOI:** 10.3389/fimmu.2022.833490

**Published:** 2022-04-04

**Authors:** Jiayue Ding, Baoying Song, Xiran Xie, Xaingyu Li, Zhiying Chen, Zhongao Wang, Liqun Pan, Duo Lan, Ran Meng

**Affiliations:** ^1^ Department of Neurology, Tianjin Medical University General Hospital, Tianjin, China; ^2^ Department of Neurology, Xuanwu Hospital, Capital Medical University, Beijing, China; ^3^ Advanced Center of Stroke, Beijing Institute for Brain Disorders, Beijing, China; ^4^ Department of Neurology, Tianjin Huanhu Hospital, Tianjin, China

**Keywords:** cerebral venous thrombosis, inflammation, brain injury, COVID-19, cytokines

## Abstract

Cerebral venous thrombosis (CVT) is a rare form of cerebrovascular disease that impairs people’s wellbeing and quality of life. Inflammation is considered to play an important role in CVT initiation and progression. Several studies have reported the important role of leukocytes, proinflammatory cytokines, and adherence molecules in the CVT-related inflammatory process. Moreover, inflammatory factors exacerbate CVT-induced brain tissue injury leading to poor prognosis. Based on clinical observations, emerging evidence shows that peripheral blood inflammatory biomarkers—especially neutrophil-to-lymphocyte ratio (NLR) and lymphocyte count—are correlated with CVT [mean difference (MD) (95%CI), 0.74 (0.11, 1.38), p = 0.02 and −0.29 (−0.51, −0.06), p = 0.01, respectively]. Moreover, increased NLR and systemic immune-inflammation index (SII) portend poor patient outcomes. Evidence accumulated since the outbreak of coronavirus disease-19 (COVID-19) indicates that COVID-19 infection and COVID-19 vaccine can induce CVT through inflammatory reactions. Given the poor understanding of the association between inflammation and CVT, many conundrums remain unsolved. Further investigations are needed to elucidate the exact relationship between inflammation and CVT in the future.

## Introduction

Cerebral venous thrombosis (CVT) is a subtype of stroke, which accounts for 0.5%–1% of all cases of stroke; it mainly occurs in young and middle-aged individuals (1). Recent studies reported a CVT incidence of 1.3–1.6/100,000 in Western countries and even higher in Asia (2, 3). CVT usually involves either dural sinuses or cortical veins and results in variable pathogenic factors and complex clinical features (2). Similar to cerebral arterial ischemic stroke, inflammation is one of the well-known risk factors for CVT onset and development. Infection or autoimmune-mediated inflammatory reaction leads to localized hyper-coagulation and endothelial injury, which may cause CVT eventually; CVT-induced inflammation further aggravates brain tissue ischemic injury and leads to poor clinical outcomes (2, 3). Therefore, inflammation is a predisposing and exacerbating factor for CVT onset and development. The diagnostic and treatment strategies for CVT cannot be separated from those for inflammation in clinical settings. This review summarized the relationship between CVT and inflammation to serve as a reference for further studies.

## Methods

**Figure 1:**
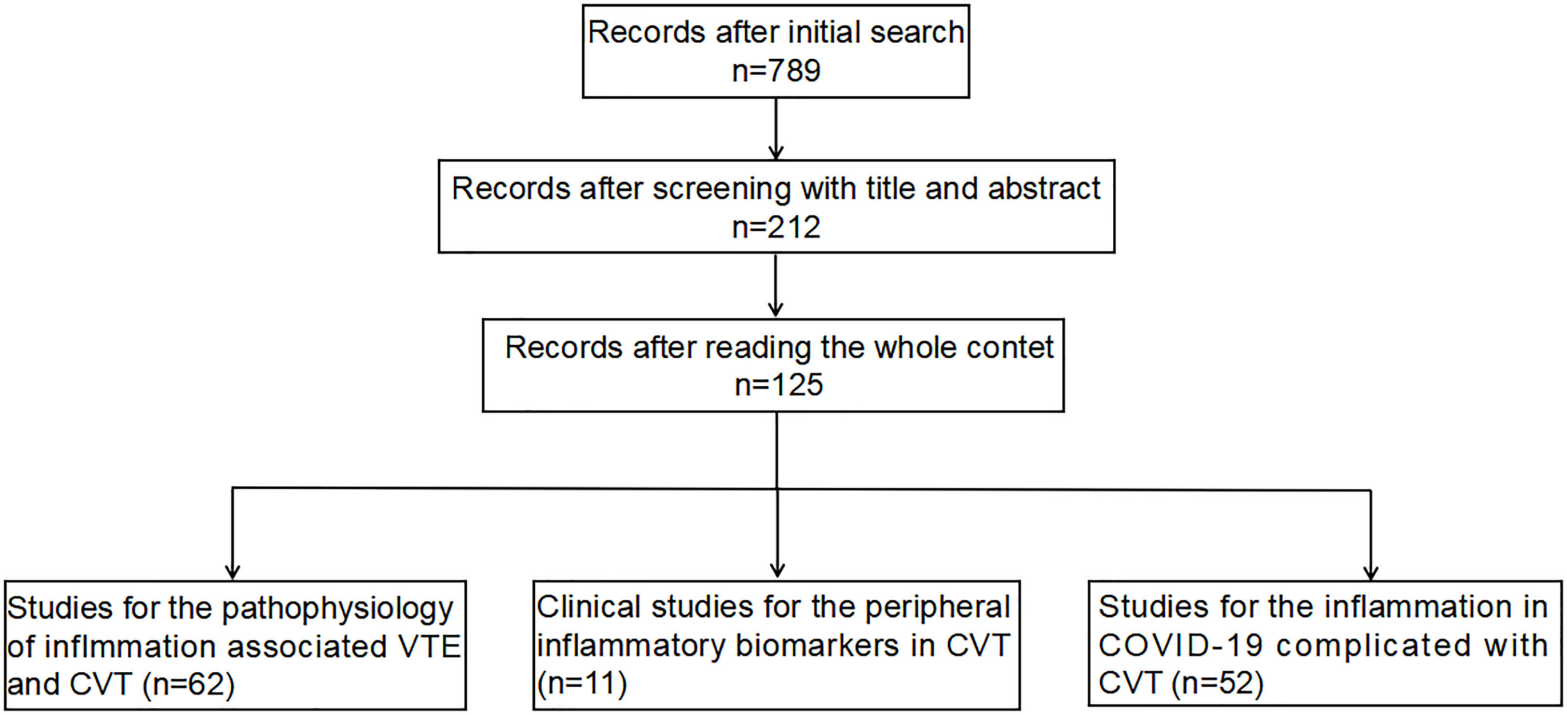
The selection flowchart for this review.

We searched several literature databases, including PubMed, Embase, and Cochrane, for publications with specific keywords, “venous thrombosis/thrombus/thrombi” and “inflammation/inflammatory” or “immune/immunology/immunological,” that were published prior to December 2021. The references were subsequently thoroughly reviewed to retrieve articles for additional reports that we may have missed out on in our search. The adopted search strategy identified 125 articles in this study, including 62 studies focusing on the pathophysiology of inflammation associated with venous thromboembolism (VTE) and CVT, 11 clinical studies investigating the peripheral inflammatory biomarkers in the patients with CVT, and 52 studies on the inflammation in COVID-19 complicated with CVT (a study selection flowchart has been shown in **Figure 1**).

The clinical studies were further conducted with meta-analysis. Stata software (version 15.0 SE) was used for analysis in this study. The values are presented as mean ± SD. Estimating the sample mean and SD from the sample size, median, and interquartile range was based on the method provided by Luo et al. and Wan et al. (4, 5). Pooled analysis was conducted with the fixed-effects model using the Mantel–Haenszel method when the heterogeneity was expected to be available (I^2^ ≤ 50%). Otherwise, the random-effects model computed through the DerSimonian–Laird method was performed (I^2^ > 50%). p-Values < 0.05 were considered statistically significant.

## Inflammation Promotes Cerebral Venous Thrombosis Onset

It is generally acknowledged that inflammation plays a central role in the occurrence of VTE, but the predisposing effect of inflammation on CVT remains poorly understood. However, known risk factors of CVT, such as infection, anticoagulant factor deficiency, and systematic autoimmune diseases, are linked with inflammatory reactions, indicating that inflammation may be involved in cerebral venous thrombogenesis (2, 3). Clinical studies that reported the conspicuous activation of serum and cerebrospinal fluid (CSF) inflammatory biomarkers in CVT further verified inflammation’s causative role in CVT onset (6–16). VTE and CVT are deemed to share similar inflammatory mechanisms clinically. Hence, the inflammatory pathogenesis in VTE seems to mimic the inflammatory reactions in CVT. The formation of venous thrombus of any type could be attributed mainly to three key elements—blood flow alteration, endothelial injury, and hypercoagulable state—collectively known as Virchow’s triad (17). Endothelial injury exposes collagen and tissue factors (TFs) that promote platelet aggravation and activate the immune system to participate in thrombus formation (18, 19). The prominent immunological factors for VTE mainly include leukocytes, cytokines, and adhesion molecules, all of which are likely to be implicated in the development of CVT (20). Given the paucity of reports on the effects of inflammation on the development of CVT, this study described the major inflammatory processes in VTE to mimic the possible inflammatory pathogenesis in CVT development.

### Leukocytes

Currently, innate immune response in VTE has attracted tremendous attention worldwide. Neutrophils, which patrol the bloodstream and contribute to pathogen clearance by phagocytosis, serve as essential leukocytes in the innate immune response. Neutrophils are responsible for the inflammatory process in venous thrombus formation, regarded as a double-edged sword during thrombosis: early massive activation resulting in tissue damage promotes thrombus propagation, whereas late activation contributes to thrombus resolution (21, 22). Neutrophils extrude DNA in the form of neutrophil extracellular traps (NETs), which play an important role in the inflammatory pathophysiology of thrombus formation (23). NETs are comprised of myeloperoxidase and histones, which stimulate fibrin formation to trap and destroy invading microorganisms. In addition, NETs directly activate the Hageman factor (FXII), bind to von Willebrand factor (vWF), trigger platelet recruitment, and increase the concentration of enzymes such as neutrophil elastase and myeloperoxidase to promote intrinsic coagulation and thrombus formation (23–25). Besides, neutrophil elastase and myeloperoxidase can cleave and oxidize anticoagulants as well, leading to thrombus extension (25). Engelmann and Massberg coined the mechanism of NET-mediated microvascular thrombosis as “immunothrombosis” (25). Immunothrombosis inhibits the spread of infection through 1) capturing and ensnaring pathogens in the microvasculature, 2) restricting pathogens movement for bactericidal activity by the action of innate immune cells, and 3) recruiting other immune cells such as monocytes and macrophages to the invaded sites for further bactericidal activity (25). Thus, immunothrombosis may be an essential contributor to venous thrombus formation. Although it has not been proven in CVT pathogenesis, we hypothesized that immunothrombosis might be responsible for infection-mediated CVT, especially cortical venous thrombosis.

Besides neutrophils, monocytes play a role in immunothrombosis by producing microparticles with TFs (26). Accepted views consider thrombus formation in the venous vasculature to be driven by TF from the vessel wall when endothelial cells are destroyed (27, 28). However, Brühl et al. found that TF expressed by Ly6G+ monocytes showed a strong signal in VTE, indicating that both vessel wall TF and blood cell-derived TF critically contribute to venous thrombosis (29). Microparticles are encrypted in the monocyte genome and are in low content in normal physical conditions (26). When stimulated by pattern recognition receptors (PRRs), microparticles within monocytes deliver the activated TF to the sites of pathogen exposure by assisting with adherence of extruded NETs, which trigger the extrinsic pathway of coagulation finally (29, 30). The synergy between neutrophil-released NETs and monocyte-released microparticles participates in thrombus formation and elongation in veins, including cerebral veins.

Mast cells are another important component of the immune system that plays a significant role in venous thrombosis (31). Mast cells exert their prothrombotic actions by releasing the granular constituent, including histamine, cytokines, and proteases, which cause endothelial activation, platelet adhesion, vWF secretion, leukocyte recruitment, P-selection release, and intercellular adhesion molecule-1 (ICAM-1) expression (31, 32). During a hypersensitivity reaction, polyphosphate (polyP) serves as a potent proinflammatory signal released from mast cells assisting with coagulation by promoting factor V activation, reducing TF pathway inhibitor (TFPI) activity, and retarding thrombolysis by eliciting thrombin-activatable fibrinolysis inhibitor (TAFI) (33–35).

**Figure 2:**
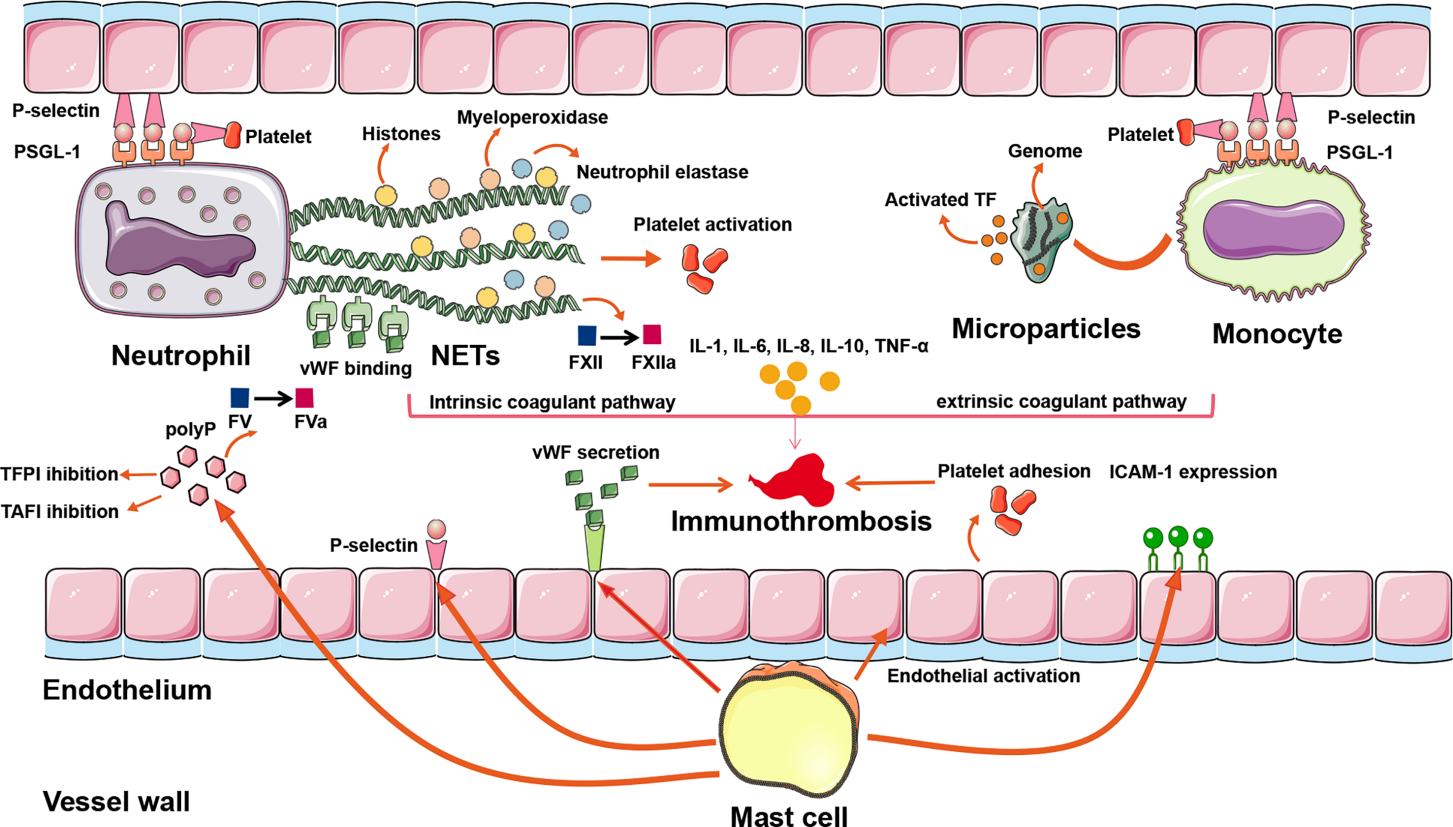
The inflammatory mechanisms of CVT formation. A plethora of inflammatory pathways are implicated in CVST formation: (1) NETs extruded by neutrophils can directly activate FXII, bind to vWF, trigger platelet recruitment, and concentrate enzymes such as neutrophil elastase and myeloperoxidase, so that intrinsic coagulant pathway is activated. (2) Activated TF is delivered by microparticles within monocytes to the sites of pathogen exposure. Assisting with adherence of extruded NETs, the extrinsic coagulant pathway is triggered. (3) Mast cells exert their prothrombotic actions through the release of a granular constituent, leading to endothelial activation, platelet adhesion, vWF secretion, leukocyte recruitment, P-selection release, and ICAM-1 expression. (4) Proinflammatory cytokines such as IL-1, IL-6, IL-8, IL-10, and TNF-α modulate the inflammatory reactions to affect the evolution or resolution of thrombosis. (5) Binding of P-selectin to PSGL-1 initiates the rolling leukocytes adhering on activated platelets and endothelial cells, renders neutrophils migrating to the sites of injury, promotes NETs formation in the setting of activated platelets, and promotes monocytes releasing TF-bearing microparticles. ICAM-1, intercellular adhesion molecule-1; NETs, neutrophil extracellular traps; polyP, polyphosphate; PSGL-1, P-selectin glycoprotein ligand 1; TAFI, thrombin-activatable fibrinolysis inhibitor; TF, tissue factor; TFPI, tissue factor pathway inhibitor; vWF, von Willebrand factor; CVT, cerebral venous thrombosis; CVST, cerebral venous sinus thrombosis.

In summary, immune cells precipitate venous thrombosis through the following pathophysiological pathways: neutrophils extrude NETs and activate FXII to FXIIa to trigger intrinsic coagulation; monocytes release microparticles that deliver TF to trigger extrinsic coagulation; and mast cells promote platelet activation and adhesion to support immunothrombosis (**Figure 2** and **Table 1**). In theory, the aforementioned mechanisms may apply to both arterial and venous thrombosis, including CVT.

**Table 1:** Proinflammatory factors implicated in venous thrombosis formation and development.

Factors	Functions
Cytokines	
IL-1	IL-1 promotes coagulation through upregulating TF expression, downregulating the expression of thrombomodulin and endothelial cell protein C receptor, increasing the production of PAI, and decreasing the production of t-PA (36–38).
IL-6	IL-6 can induce the expression of TF, fibrinogen, factor VIII, and vWF and lower the concentration of antithrombin, protein S, and thrombomodulin (39, 40).
IL-8	IL-8 is the prototypical member of CXC ELR+ chemokines that are generally neutrophil-attractant and proangiogenic. It can enhance venous thrombosis resolution (41).
IL-10	IL-10 is capable of inhibiting the activation of T cells, monocytes, and macrophages, as well as downregulating the expression of P-selectin. IL-10 restrains inflammatory events and inhibits thrombus formation finally (42–44).
IL-18	IL-18 can alter NF-κB, causing damage, apoptosis, and other changes of the venous endothelial cells. The changes of endothelial cells cause anomalies in thrombotic disease-related cell function markers, vWF, P-selectin, and t-PA, resulting in thrombus formation (45).
TNF-α	TNF-α can upregulate TF’s expression and promote venous thrombus formation (46).
IFN-γ	IFN-γ can induce the formation of NETs by neutrophils with subsequent venous thrombosis development and delay thrombus resolution by inhibiting MMP-9 production by monocytes (47, 48).
CCR2/CCL2	CCR2/CCL2 is the main chemokine and chemokine receptor that is involved in the recruitment of monocytes in vascular disease. CCR2 is required for thrombin-induced monocyte migration, and CCL2 secreted by endothelial cells contributes to an increase of monocyte migration and PSGL-1 expression (49).
Cellular markers	
CD40L	The binding of CD40L to its CD40 receptor on the leukocyte can enhance TF expression, induce endothelial cells to recruit leukocytes, and promote thrombin generation through overexpression of TF (50).
CD39	CD39 protects from venous thrombogenesis in restricted venous flow conditions by checking leukocyte engagement, suppressing inflammasome activation, and restraining innate immune activation finally (51).
Adhesion molecules	
P-selectin	P-selectin stored in endothelial cells and platelets binds to PSGL-1 presented on the surface of leukocytes, resulting in secretion of TF-bearing MPs from leukocytes and subsequently initiating procoagulatory mechanisms. Signaling through P-selectin by PSGL-1 can also assist in PMN migration to the sites of inflammation and promote NETosis (52–56).
Integrin	Integrin β2 expressed on the neutrophils and integrin β3 expressed on the platelets with ligand fibrinogen are directly involved in the formation of acute venous thrombosis (57).
Leukocyte integrin Mac-1	Leukocyte integrin Mac-1 acts as a prerequisite for thrombosis initiation through mediating adhesion of leukocyte–endothelium *via* ICAM-1 and adhesion of platelets by GPIba (58).
CLEC-2	CLEC-2 is a receptor for podoplanin released from the endothelium and triggers thrombosis formation (59).
Complements	
C3a and C5a	C3a and C5a support immunothrombosis by triggering platelet activation (60).
Complex	
NETs	NETs are extruded from neutrophils and contain myeloperoxidase, enzymes, and histones. These NETs can activate FXII leading to the initiation of the intrinsic pathway, bind to TF leading to the activation of the extrinsic pathway, release histones to produce thrombin by platelets, release vWF leading to platelet and leukocyte adhesion, and locally concentrate enzymes such as neutrophil elastase and myeloperoxidase that cleave and oxidize anticoagulants, respectively (23–25).
MPs	MPs with TF are small, phospholipid vesicles shed from monocytes stimulated by PRRs. MPs can deliver activated TF leading to the extrinsic pathway activity, interact with platelets and endothelium mediated by PSGL-1 on the microparticles and P-selectin on the platelets and endothelium, and inhibit fibrinolysis (29, 30).
Inflammasomes	NLRP3 inflammasomes are molecular complexes primarily concentrating on the transformation of caspase-1 and caspase-11 into their active forms, which leads to cleavage and activation of IL-1β and IL-18. IL-1β stimulates TF’s release associated with NETs. A cross-talk between NETs and inflammasomes promotes venous thrombosis formation (61).
Enzymes	
PAD4	PAD4 is an enzyme essential for the citrullination and decondensation of chromatin. It participates in NET formation and facilitates thrombus generation (62).
PMN elastase	PMN elastase can colocalize with NETs (23–25).
Serine proteases	Serine proteases can colocalize with NETs and inhibit TFPI to further induce thrombosis (63).
PDI	PDI can promote thrombus formation by facilitating platelet accumulation and participating with neutrophils in TF activation (64).
MMPs	MMPs regulate inflammatory mediators during venous thrombus resolution and reduce vessel wall fibrosis (65).
Sirt3	Decline of Sirt3 activity leading to intracellular ROS’s elevation can alter neutrophil and platelet function, resulting in enhancement of thrombosis (66).
Other molecules	
Poly-P	Poly-P is released from platelet-dense granules upon platelet activation, and it is also released from mast cells during a hypersensitivity reaction. It can activate factor V, decrease TFPI activity, and stimulate TAFI activity (33–35).
HMGB-1	HMGB-1 is released from the damaged cells and expressed by the activated platelets, leading to immune system activation *via* RAGE, TLR2, and TLR4. HMGB-1-mediated PMN activation subsequently contributes to microvascular thrombosis and NETosis (67, 68).
Gas6	Gas6 can amplify endothelial cell activation through TF expression, collect platelets and leukocytes to the endothelial cell membrane, and promote the recruitment of monocytes through a CCR2/CCL2-dependent mechanism during venous thrombosis (49).
CRP	CRP can stimulate platelet adhesion and responsiveness, promote P-selectin expression on the surface of endothelial cells, increase TF expression, and decrease TFPI expression, so as to accelerate venous thrombus growth (69).
TLR9	TLR9 is a conserved PAMP and DAMP receptor that alerts the immune system to invading pathogens or local damage. It can decrease citrullinated histones, PAD4, and neutrophil elastase and increase TFPI, so as to induce thrombosis resolution (70).

IL,: interleukin; TF,: tissue factor; PAI, plasminogen activator inhibitor; t-PA, tissue plasminogen activator; vWF, von Willebrand factor; TNF, tumor necrosis factor; IFN, interferon; CCR2, chemokine receptor type 2; CCL2, chemokine ligand 2; PSGL-1, P-selectin glycoprotein ligand-1; MPs, microparticles; PMN, neutrophil; NETs, neutrophil extracellular traps; ICAM-1, intracellular adhesion molecule-1; CLEC-2, platelet C-type lectin-like receptor; PRRs, pattern recognition receptors; PAD4, peptidyl arginine deiminase 4; TFPI, tissue factor pathway inhibitor; PDI, protein disulfide isomerase; MMPs, matrix metalloproteases; ROS, reactive oxygen species; Poly-P, polyphosphate; TAFI, thrombin-activatable fibrinolysis inhibitor; HMGB-1, high-mobility group box protein 1; RAGE, receptor for advanced glycation end-products; TLR, toll-like receptor; Gas6, growth arrest-–specific 6; CRP, C-reaction protein; PAMP, pathogen-associated molecular pattern; DAMP, damage-associated molecular pattern.

### Proinflammatory Cytokines

Varieties of proinflammatory cytokines, such as interleukin-6 (IL-6), interleukin-1 (IL-1), interleukin-8 (IL-8), and tumor necrosis factor-alpha (TNF-α), are involved in venous thrombus formation. They modulate the inflammatory reactions to affect the development or resolution of thrombosis and play an essential role in thrombosis by promoting TF expression resulting in a procoagulant state (46, 71, 72). IL-6 is the most notable cytokine in boosting coagulation and has been used to evaluate the coagulant status and predict the clinical outcomes of CVT patients (7, 12, 73). A high level of IL-6 in VTE is strongly correlated with a high level of fibrinogen, high rates of complications and thrombosis recurrence, and poor clinical outcomes (7, 12, 74, 75). IL-6 increases the expression of TF, fibrinogen, factor VIII, and vWF, leading to endothelial cells activation, vessel wall damage, platelet aggregation, and recruitment and activation of leukocytes at the venous wall, all of which provoke the localized thrombus formation (39, 40). Additionally, IL-6 can reduce the concentration of the natural thrombosis inhibitors, such as protein S, antithrombin, and thrombomodulin, resulting in thrombus formation (40). Thus, IL-6 serves as an important promoter of thrombosis in VTE. Other risk factors for VTE mainly include IL-1, IL-8, IL-18, TNF-α, IFN-γ, and CCR2/CCL2, and their functions are shown in **Table 1**.

In contrast, IL-10 plays a beneficial role in patients with VTE by its downregulatory effect on the immune system (42). IL-10 is deemed an anti-inflammatory cytokine capable of inhibiting the activation of T cells, monocytes, and macrophages (43). In addition, P-selectin stored in platelets and endothelial cells is also significantly suppressed by IL-10 (44). Therefore, IL-10 restrains inflammatory events and inhibits thrombus formation. Although elevated proinflammatory cytokines’ beneficial or detrimental influences on CVT are uncertain, the indispensable role of cytokines in venous thrombus formation has been well-acknowledged (**Figure 2** and **Table 1**).

### Adhesion Molecules

Adhesion molecules are a critical component implicated in the inflammatory process and thrombus generation. P-selectin is one of the hot topic adhesion molecules for VTE pathogenesis and serves as an interaction mediator among platelets, leukocytes, and endothelial cells that modulate both hemostasis and inflammation (52). P-selectin is an extended protein with a membrane-distal C-type lection domain, always bonded to P-selectin glycoprotein ligand 1 (PSGL-1) mainly expressed in the neutrophils and monocytes (53). Endothelial cells and platelets activated by thrombin or other mediators rapidly mobilize P-selectin from secretory granules to the cell surface when infection or injury occurs (53, 54). The binding of P-selectin to PSGL-1 initiates the rolling leukocytes’ adhesion to activated platelets and endothelial cells and engenders platelets’ microparticles to form bridges among leukocytes (54). In addition, signaling by P-selectin through PSGL-1 aids neutrophils’ migration to the sites of injury and promotes NET formation in the setting of activated platelets (55, 56). Likewise, P-selectin can promote monocytes releasing TF-bearing microparticles, resulting in extrinsic coagulation initiation (29, 76). When blocking the binding of P-selectin to PSGL-1, leukocyte adhesion to platelets and endothelial cells is inhibited, and the expression of molecules that amplifies inflammation and thrombosis is also suppressed (76–79). Herein, P-selection is considered as an important mediator implicated in venous immune-related thrombosis and aggravation (**Figure 2**). Other adhesion molecules such as integrin, leukocyte integrin Mac-1, and CLEC-2 also play an important role in VTE initiation, shown in **Table 1**.

### Summary

The factors mentioned above have been fully elucidated and are considered the major inflammatory reactions involved in VTE. Additionally, a growing body of proinflammatory factors such as complements, cellular markers, and secretory proteins is also implicated in venous thrombosis (**Table 1**) (23–25, 29, 30, 33–70). The processes are complex and varied, and many mechanisms remain poorly understood. Given that inflammation is essential in venous thrombus formation, inflammatory diseases that produce high immune system activation are definite risk factors for CVT. Usually, the incidence of VTE in patients with inflammatory diseases is threefold higher than in general populations (80). In particular, some acquired, multi-organ, inflammatory diseases such as systemic lupus erythematosus (SLE), Behçet’s disease (BD), and inflammatory bowel disease (IBD) are independent risk factors for CVT (81–83). Both central and peripheral inflammatory markers such as NETs, TF, P-selectin, IL, and C-reactive protein (CRP) are considered to be mediated by inflammatory diseases and accompany CVT onset and aggravation (84–87). In patients with inflammatory disease-related CVT, inflammatory reaction control cannot be overlooked. In these settings, immunosuppressives combined with anticoagulant therapies will be suggested. With the cooperation of leukocytes, cytokines, adhesion molecules, and other factors, the inflammation affects the onset and development of venous thrombosis and can be considered a paramount issue for CVT onset.

## Inflammation in Cerebral Venous Thrombosis-Related Brain Damage

**Figure 3:**
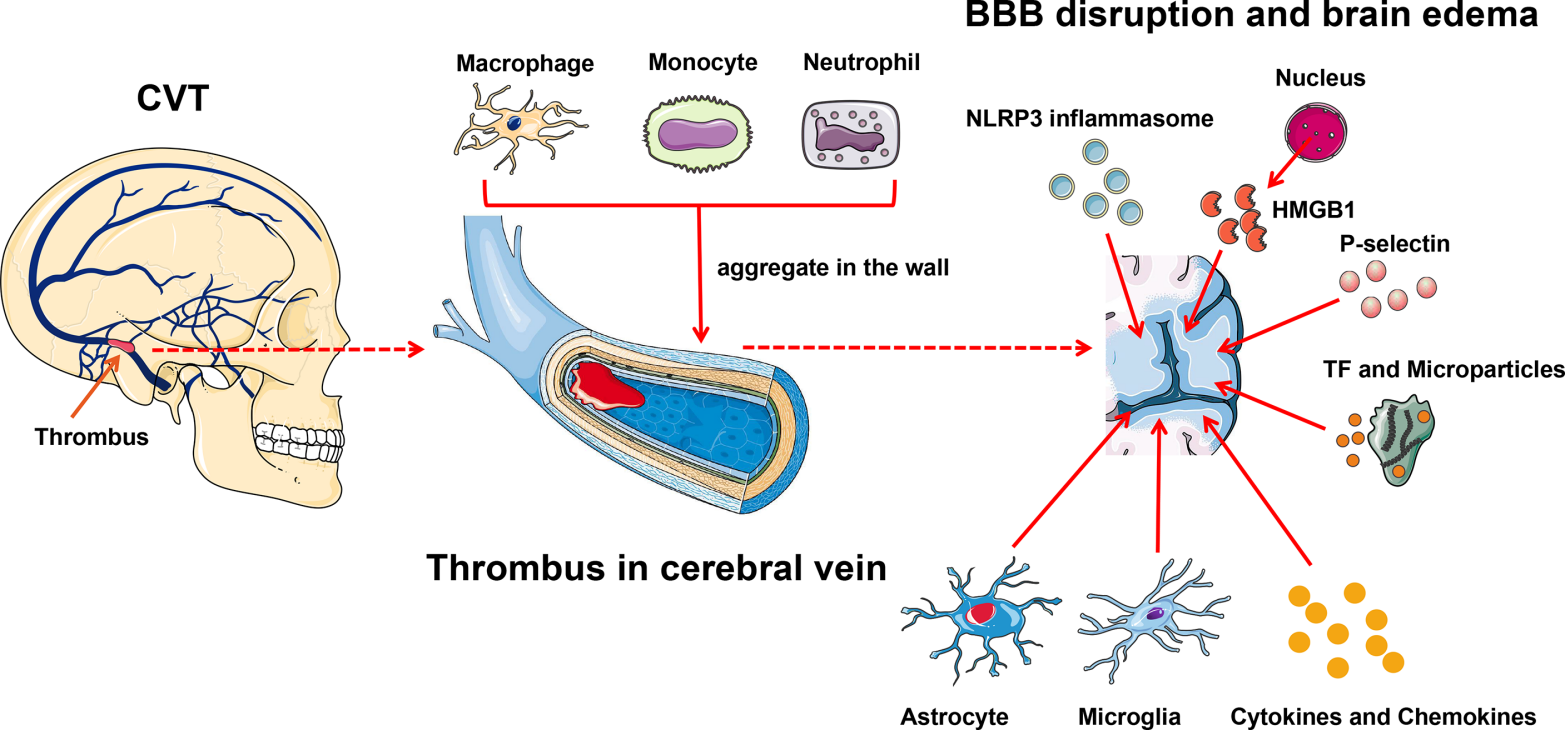
Inflammation exacerbates CVT-induced brain injury. Leukocyte–endothelial cell adhesion contributes to brain damage after CVT. Initially, neutrophils aggregate in the venous wall followed by monocytes and macrophages; meanwhile, microglia and astrocyte infiltrate into the damaged tissue. And then, cytokines, chemokines, and other inflammatory factors such as TNF-α are released to elicit an inflammatory reaction. P-selectin and activated TF within microparticles further amplify the brain tissue injury. NLRP3 is involved in the inflammatory reaction post-CVT through eliciting caspase-1 to cleave pro-IL-1β and pro-IL-18 to their active forms. HMGB1, which is released from the nucleus, can induce endocytosis and generate proinflammatory factors to further damage the brain tissue. BBB, blood–brain barrier; CVT, cerebral venous thrombosis; HMGB1, high-mobility group box 1; TF, tissue factor.

Similar to cerebral arterial infarction, an inflammatory reaction also occurs after a CVT attack (**Figure 3**). Blood–brain barrier (BBB) disruption inducing vasogenic edema is a pivotal pathological phenomenon after CVT, and leukocyte–endothelial cell adhesion contributes to this process (88). Nagai et al. reported that no BBB disruption or brain edema was detected 3 h post-CVT; however, increased leukocyte adhesion, elevated monocyte chemotactic protein 1 (MCP-1), and decreased IL-10 were observed, suggesting that inflammation might occur ahead of the edema (89). At hour 48 after CVT, the abnormal elevated IL-10 was restored, MCP-1 level remained high, and BBB disruption and edema were noted (89). On day 3 post-CVT, T-cell recruitment, microglia and macrophage activation, and astrocyte aggregation could be seen, and these phenomena were maintained for 1 to 2 weeks (88). Initially, neutrophils aggregate in the venous walls, followed by monocytes and macrophages. Then, cytokines, chemokines, and other inflammatory factors such as TNF-α elicit inflammatory reactions (90). Proinflammation and anti-inflammation are jointly mediated by the venous wall damage ultimately. P-selectin accelerates the inflammatory reaction in this process, and the deletion of P-selectin profoundly inhibits the venous wall inflammatory response (44, 91, 92). In addition, microparticles and TF further amplify the inflammatory reaction and promote thrombus growth and tissue damage (28).

NLRP3, the most characterized inflammasome, is found abundantly in the brain (61, 88, 93). The role of NLRP3 in CVT is well documented. Unlike in arterial ischemia, where NLRP3 changes suddenly minutes to hours after stroke onset, NLRP3 in CVT activates in a subacute fashion, evident at day 3, and maintained until day 7 after CVT, which is in parallel with the immune cell activation and infiltration (88, 94). Thioredoxin-interacting protein (TXNIP) is a well-known binding partner to NLRP3 and is responsible for NLRP3 inflammasome activation under endoplasmic reticulum (ER) stress and oxidative stress (95, 96). ER stress and oxidative stress jointly boost the expression and activation of TXNIP (94). Meanwhile, peroxynitrite formation, which is elicited by oxidative stress, is also a pivotal trigger of NLRP3 inflammasome activation (94, 97). Therefore, TXNIP and peroxynitrite jointly facilitate the activation of NLRP3 inflammasome after CVT under ER stress and oxidative stress. NLRP3 can elicit caspase-1 to cleave pro-IL-1β and pro-IL-18 to their active forms, both of which are involved in the inflammatory reaction post-CVT (93, 98). Meanwhile, NLRP3 can lead to pyroptosis after CVT, validated by gasdermin D (GSDMD, an indicator of pyroptosis) (94).

High mobility group box 1 (HMGB1), a member of the damage-associated molecular pattern molecular family, is recently reported to be implicated in the inflammatory cascade-amplification reaction in the pathophysiology of CVT (67). Under normal physical conditions, HMGB1 exists in the nucleus and maintains the chromosomal structure and physiological activities of the DNA (99). On suffering inflammatory stimuli, HMGB1 is released into the extracellular space and immediately activates the innate immune response (99). Independently, HMBG1 can activate its downstream mediator, the receptor for advanced glycated end products (RAGE), to induce endocytosis and generate proinflammatory factors such as TNF-α, IL-1β, and IL-6 (67). The inhibition of HMGB1 and RAGE can reduce the expression of downstream inflammatory factors and attenuate CVT-mediated further damage (67).

Although the understanding is far from adequate, the pivotal role of inflammation on CVT-related brain tissue damage is undoubted. Currently, how to inhibit post-CVT inflammation progression presents a great interest in CVT correction. Since immunosuppressors have been gradually used in cerebral arterial ischemia, the inhibition of immunological reactions may be a novel promising strategy for CVT treatment (100).

## Peripheral Inflammatory Biomarkers for Cerebral Venous Thrombosis

Several reports support the central role played by the inflammatory process in thrombosis development. Besides the focal inflammatory response described earlier, peripheral inflammatory markers also correlate with the pathogenesis of thrombosis. Peripheral neutrophils and lymphocytes are strongly associated with the severity and prognosis of cerebral arterial ischemia (101). Similarly, it is believed that the inflammatory parameters are also altered in CVT (7). Elevated inflammatory parameters can reflect the severity and predict the prognosis of CVT.

### Peripheral Inflammatory Biomarkers Are Characterized in Cerebral Venous Thrombosis

**Figure 4:**
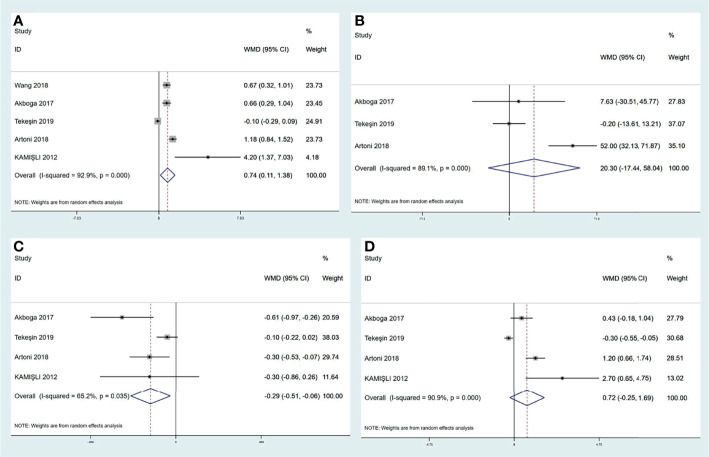
Meta-analysis of inflammatory biomarkers in CVT versus healthy controls, including NLR **(A)**, PLR **(B)**, lymphocyte count **(C)**, and neutrophil count **(D)**. CVT, cerebral venous thrombosis; NLR, neutrophil-to-lymphocyte ratio; PLR, platelet-to-lymphocyte ratio.

A variety of peripheral inflammatory markers are associated with central venous thrombosis. The high neutrophil-to-lymphocyte ratio (NLR) is identified as the conspicuous index for cerebral arterial ischemia. However, the role of NLR in CVT is not clear. Four studies reported that CVT patients had a significantly elevated NLR value than controls (6, 7, 13, 16). However, a large retrospective study reported by Artoni et al., including 100 CVT patients and 299 controls, found no difference in NLR (15). We performed a pooled analysis in this review (shown in **Figure 4A**) and found that CVT patients had a higher level of NLR than controls [mean difference (MD) (95%CI), 0.74 (0.11, 1.38), p = 0.02]. Although our result conflicted with that of Artoni et al., we believe that NLR could be used as an assistant biomarker for CVT evaluation. Platelet-to-lymphocyte ratio (PLR) is also a predictive value for cerebral arterial ischemia (101). Two studies demonstrated a statistical correlation between higher PLR value and CVT in relation to cerebral arterial ischemia (6, 13). In contrast, Artoni et al. showed a lack of association between them (15). When pooling these results together (shown in **Figure 4B**), no significant difference in PLR values was obtained between CVT patients and controls [20.30 (−17.44, 58.04), p = 0.29]. Hence, PLR might not be suitable for CVT diagnosis. Neutrophil and lymphocyte counts were also analyzed in previous studies: Artoni et al. found higher neutrophil count in controls than in CVT patients (15). In contrast, three other studies did not find a statistical difference in neutrophil count between the two populations (6, 13, 16). Intriguingly, the 3 studies found significantly lower lymphocyte counts in CVT patients than in controls, but Artoni et al. did not (6, 13, 15, 16). The results regarding neutrophil and lymphocyte counts were also pooled together in this review. As shown in **Figures 4C, D**, lymphocyte count was significantly lower in CVT patients than controls [−0.29 (−0.51, −0.06), p = 0.01], but the neutrophil count [0.72 (−0.25, 1.69), p = 0.14] did not show a significant difference between the groups. In addition, pooled analysis for platelet count from the three studies did not find any difference between the 2 groups [20.30 (−17.44, 58.04), p = 0.29] (6, 13, 15). All results mentioned above uncovered the critical role of lymphocyte count as a laboratory feature for CVT. Although neutrophils and platelets were responsible for venous thrombus initiation and development, this review considered the possible characterization of NLR value and lymphocyte count with CVT.

### Peripheral Inflammatory Biomarkers Predict the Prognosis of Cerebral Venous Thrombosis

Inflammation aggravates CVT-related brain injury resulting from various etiologies, leading to poor prognosis. Except for leukocyte infiltrating into the focal lesion, peripheral leukocyte variations are more convenient to be detected in clinical settings. Since the NLR value is the most important inflammatory index characterized by CVT, it also seems to predict CVT’s prognosis. Wang et al. found that increased serum NLR value was associated with the severity of CVT patients at admission and reflected the short-term outcome at discharge (7). Sousa et al. indicated that increased serum NLR value had a significant association with the unfavorable functional outcome at 90 days (9). Apart from NLR, other inflammatory marker variations such as low serum lymphocyte-to-monocyte ratio (LMR), high serum IL-6 concentration, and high CSF IgM concentration correlate with poor outcomes of patients with CVT (7, 9, 11). To comprehensively reflect the balance of host immune and inflammatory status in CVT, Li et al. used systemic immune-inflammation index (SII) to predict the prognosis of patients with CVT. SII is defined as follows: platelet (/L) × neutrophil (/L)/lymphocyte (/L) (8). They demonstrated that higher SII presented lower survival rates and was a significant predictor of poor outcomes. Neutrophils are considered an important immune cell facilitating venous thrombus formation, and lymphocytes are also involved in the inflammatory process of developing blood clots (102). Given the interaction among neutrophils, lymphocytes, and platelets, SII may be more available to evaluate the inflammatory extent during CVT.

### Peripheral Inflammation Biomarkers Fluctuate in Different Cerebral Venous Thrombosis Phases

Peripheral inflammation markers are different among acute, subacute, and chronic CVT phases. The acute and subacute phases of CVT have a higher neutrophil count, lower NLR value, and higher CRP levels than the chronic phase (7, 14). In addition, lower lymphocyte counts and higher NLR values are observed in patients with acute onset CVT than those with subacute and chronic symptoms (7, 14). Intriguingly, the monocyte count is higher in the subacute phase than in the chronic phase, but there is no significant difference between the acute and chronic phases (14). Antibodies in CSF such as IgM, IgA, and IgG are substantially lower in the chronic phase than in the acute and subacute phases (7). All results mentioned above indicate the inflammatory activity peaks at the acute phase, which is the most severe stage, decreases gradually at the subsequent phases, and returns close to normal at last.

Overall, this part indicates that peripheral inflammatory biomarkers are invariably associated with CVT progress and prognosis in clinical settings. The prominent markers such as NLR, lymphocyte count, and SII are widely applied to CVT diagnosis, monitoring, and evaluation of treatment. However, the variations in peripheral inflammatory biomarkers as either a contributor to or result of CVT may be less clear. Thus, the connections between central damage and peripheral inflammation are necessary to be investigated in the future.

## COVID-19 Complicated With Cerebral Venous Thrombosis

### COVID-19 Infection-Induced Cerebral Venous Thrombosis

The novel severe acute respiratory syndrome coronavirus-2 [SARS-CoV-2, also named coronavirus disease-19 (COVID-19)] pandemic currently challenges the global public health and healthcare sectors. The complications and prognosis of COVID-19 are manifold. Clinical observations show an increased risk of stroke and thrombotic complications in patients with COVID-19 infection (103). COVID-19 infection itself has a rare but demonstrated association with CVT. Inflammation is also implicated in this disease’s progression. A recent systematic review using data from 34,331 patients identified the frequency of CVT among patients hospitalized for COVID-19 infection to be 0.08%, which is potentially higher than the expected incidence of 5 to 20 per million per year in the general population (104). Besides, two retrospective studies also reported higher incidence rates of CVT in patients with COVID-19 infection than in the general population (2–39 patients per million versus 0.4 per million) (105, 106).

The underlying mechanisms of COVID-19 infection-induced CVT are complex. Inflammatory reactions contribute to this process substantially. Vascular endothelial dysfunction caused by COVID-19 infection is the most accepted pathophysiology to elucidate the observed high incidence of CVT (107). It has been documented that both the virion and the angiotensin-converting enzyme 2 receptors are found in endothelial cells (108, 109). Besides, recruitment of immune cells, either by the viral infection of the endothelium or immune-mediated, causes endothelial dysfunction (110). The dysfunctional endothelium releases vWF and exposes locally provided TF, consequently activating the extrinsic pathway (110). COVID-19 infection-induced endothelial dysfunction causes hypercoagulability and fibrinolysis shutdown, as evidenced by elevated D-dimer and complete failure of clot lysis on thromboelastography (111). In addition, excessive activity of cytokine, coined as “cytokine storm” with high concentrations of proinflammatory cytokines and chemokines (such as IL-1, IL-6, and TNF-α), can suppress anticoagulant systems, release vWF, and increase TF expression in the backdrop of COVID-19 infection (112, 113). Neutrophils with NETs, monocytes with microparticles, and complement systems are subsequently activated, predisposing the vessels to thrombus formation and propagation (114). As Virchow’s triad criteria are fulfilled in critically ill COVID-19 patients; other pathophysiologies such as altered flow dynamics, prothrombotic and fibrinolytic imbalance, and hypoxia-mediated vasoconstriction are also responsible for CVT initiation and development (114). Therefore, CVT in COVID-19 disease is a complex interplay between the endothelium and a range of inflammatory factors.

### COVID-19 Vaccination-Induced Cerebral Venous Thrombosis

Apart from COVID-19 infection, the COVID-19 vaccine is also associated with CVT through immune thrombotic thrombocytopenia (115). One study from Germany estimated the incidence rate of CVT within 1 month after first dose administration of 1.52 per 100,000 person-months for ChAdOx1 vaccine (AstraZeneca) and 0.11 per 100,000 person-months for BNT162b2 (BioNTech/Pfizer) (116). Most of the patients had platelet-activating antibodies directed against platelet factor 4 (PF4), leading to prothrombotic status and thrombocytopenia. Similar to the pathophysiology of heparin‐induced thrombocytopenia (HIT), in the cases of vaccine-induced immune thrombotic thrombocytopenia (VITT), an unidentified polyanion in the adenoviral vaccines or expressed by the infected cells by the vaccine is likely binding to PF4 (115, 117). The complex crosslinks many FcγRIIa receptors on platelets, conducing thromboxane synthesis and platelet activation. More PF4 molecules and procoagulant microparticles are released from activated platelets, resulting in increased thrombin generation (118). In addition, increased expression of TF and NETs enhances coagulation by providing a scaffold for platelets, vWF, and red blood cells (118). The activated platelets and antibody-coated platelets are removed by the macrophages of the reticuloendothelial system, resulting in thrombocytopenia (117). In general, the COVID-19 vaccine can cause VITT, which initiates inflammatory reactions and contributes to the occurrence of CVT in patients with COVID-19 infection. Although both HIT and VITT present with antibodies to PF4, these thrombotic manifestations occur in VITT and not typically in HIT, a phenomenon yet to be explained (119).

## Conclusions

For the first time, the pivotal role of inflammation in CVT was systematically recapitulated in this review. Mounting clinical and laboratory evidence has unraveled the complex interplay among inflammation, coagulant system, and venous thrombus formation. Assisting platelets and collagen, cells involved in innate immunity, such as ILs, leukocytes, adhesion molecules, and chemokines, activate intrinsic and extrinsic coagulation pathways, leading to CVT. Innate immunity also promotes thrombus propagation and accelerates the progression of CVT. Peripheral inflammatory biomarkers may serve as convenient and accurate indices to evaluate CVT severity and patient prognosis. Exploring the inflammatory background in time for the patients with CVT will be very useful for clinicians to make a proper individualized treatment strategy for the patients. In addition, COVID-19 infection and COVID-19 vaccine administration can also induce CVT through inflammatory reactions. Due to a poor understanding of inflammation with CVT, a plethora of conundrums are yet to be resolved. Further clinical and basic studies are needed to explore and establish the exact association between inflammation and CVT in the future.

## Author Contributions

JD and RM formulated the conception of the review, drafted the manuscript, and prepared the figures. BS, XX, and XL completed the screening of the publications. ZW, LP, and DL were responsible for the statistical analysis. ZC made critical revisions to the manuscript. All authors listed have made a substantial, direct, and intellectual contribution to the work and approved it for publication.

## Funding

This study was sponsored by the National Key R&D Program of China (2017YFC1308401), the National Natural Science Foundation of China (82171297), and the Beijing Natural Science Foundation (7212047).

## Conflict of Interest

The authors declare that the research was conducted in the absence of any commercial or financial relationships that could be construed as a potential conflict of interest.

## Publisher’s Note

All claims expressed in this article are solely those of the authors and do not necessarily represent those of their affiliated organizations, or those of the publisher, the editors and the reviewers. Any product that may be evaluated in this article, or claim that may be made by its manufacturer, is not guaranteed or endorsed by the publisher.
